# Cyanobacteria Application Ameliorates Floral Traits and Outcrossing Rate in Diverse Rice Cytoplasmic Male Sterile Lines

**DOI:** 10.3390/plants11243411

**Published:** 2022-12-07

**Authors:** Hassan Sh. Hamad, Eman M. Bleih, Elsayed E. Gewaily, Ahmed E. Abou Elataa, Heba A. El Sherbiny, Noha M. Abdelhameid, Medhat Rehan

**Affiliations:** 1 Rice Research and Training Department, Field Crops Research Institute, Agricultural Research Center, Kafrelsheikh 33717, Egypt; 2 Soil Fertility and Microbiology Department, Desert Research Center (DRC), Cairo 11753, Egypt; 3 Department of Plant Production and Protection, College of Agriculture and Veterinary Medicine, Qassim University, Buraydah 51452, Saudi Arabia; 4 Department of Genetics, Faculty of Agriculture, Kafrelsheikh University, Kafr El-Sheikh 33516, Egypt

**Keywords:** cyanobacteria, hybrid rice, CMS lines, floral traits, seed yield, principal component analysis

## Abstract

In rice, cytoplasmic male sterility (CMS) represents an irreplaceable strategy for producing high-yielding hybrid rice based on the commercial exploitation of heterosis. Thereupon, enhancing floral traits and outcrossing rates in CMS lines increase hybrid seed production and ensure global food security. The exogenous application of cyanobacteria could enhance outcrossing rates in CMS lines and, accordingly, hybrid rice seed production. In the present study, we aimed at exploring the impact of cyanobacteria implementation such as *Anabaena oryzae*, *Nostoc muscorum*, and their mixture to promote the floral traits, outcrossing rates, and seed production in hybrid rice. The impact of cyanobacteria (*Anabaena Oryza* (T2), *Nostoc muscorum* (T3), and their combination (T4) versus the untreated control (T1) was investigated for two years on the growth, floral, and yield traits of five diverse CMS lines, namely IR69625A (L1), IR58025A (L2), IR70368A (L3), G46A (L4), and K17A(L5). The evaluated CMS lines exhibited significant differences in all measured floral traits (days to heading (DTH), total stigma length (TSL), stigma width (SW), duration of spikelet opening (DSO), spikelet opening angle (SOA)). Additionally, L4 displayed the uppermost total stigma length and stigma width, whereas L1 and L5 recorded the best duration of spikelet opening and spikelet opening angle. Notably, these mentioned CMS lines exhibited the highest plant growth and yield traits, particularly under T4 treatment. Strong positive relationships were distinguished between the duration of the spikelet opening, panicle exertion, panicle weight, seed set, grain yield, total stigma length, spikelet opening angle, stigma width, and number of fertile panicles per hill. Cyanobacteria is a potential promising tool to increase floral traits and seed production in hybrid rice.

## 1. Introduction

Rice (*Oryza sativa* L.) is a very important edible starchy and staple cereal food crop, as more than one half of the world’s population (>3.5 billion) depends on rice for their consumption and livelihood [1,2]. The Food and Agriculture Organization (FAO) estimates that, by 2029, the world’s food production will have to increase by 50% [3]. Thus, doubling rice production by 2050 is urgently needed to feed more than 9 billion people globally [3,4]. The productivity of released cultivars has plateaued due to narrow genetic distance in the parental lines applied in breeding programs [5], and hybrid rice is an important strategy to overcome this plateau. Hybrid rice technology provides an advantage in yield up to 20% higher than the best regular inbred rice varieties, due to a higher harvest index at the maturity stage and the accumulation of more plant biomass before flowering [6].

Large-scale cultivated hybrid rice is limited due to low amounts of hybrid seed and the high cost of seed production. To overcome this constraint, it is necessary to enhance the outcrossing ability of CMS lines for faster and wider hybrid rice adoption [7,8]. Applying anther indehiscence with exerted stigmas was hindered by trait fixation and propagation in hybrid rice breeding. The exerted stigma of rice seems to improve the outcrossing rate of the sterile lines [9,10,11]. The outcrossing in CMS lines is significantly increased by improving the floral traits, such as a longer duration of the floret opening, a longer stigma, a wider angle of the floret opening, and an exertion of higher stigma [8]. Consequently, in hybrid rice breeding programs, the floral traits of CMS lines are crucial and important. Moreover, yield traits are essential for ensuring high yield heterosis. In China, success in breeding and growing hybrid rice commercially has attracted the awareness of rice breeders worldwide [12]. In Egypt, breeding a high-yield hybrid rice is considered one of the most promising potential strategies for increasing rice production to cope with population growth [12]. Several factors influence multiplication cytoplasmic male sterile (CMS) and hybrid seed production, such as seeding time, field conditions, planting pattern, weather conditions at flowering, the synchronization of flowering between the parental lines, and supplementary pollination techniques. These practices can regulate panicle exertion from the flag leaf, enhance the rate of stigma exertion, adjust plant height, promote the duration of the floret opening, and enhance the length and productivity of late developing tillers [13,14].

Sustainable agricultural development needs novel tools for producing safe food with minimum environmental pollution. One of these is the use of plant growth promoting bacteria (PGPB), which are defined as free-living soil rhizosphere microbes. These bacteria have a good influence on both the rhizosphere and phyllosphere of the plants [15].

Cyanobacteria (blue-green algae and a Gram-negative bacteria) are an ancient group of photosynthetic microorganisms and contribute to oxygen production in the Earth’s atmosphere [16]. They are beneficial microbes found in various environments, including soil, oceans, bare rocks, and freshwater [17]. Cyanobacteria have the ability to fix atmospheric N2, solubilize phosphate, produce plant growth regulators, suppress the growth of some pathogenic microbes in soil, catalyze the nutrient cycling, produce some bioactive compounds such as vitamins and hormones that contribute to plant growth, decompose organic wastes, and detoxify pesticides, heavy metals, and other xenobiotics [18]. These microorganisms adapt to environmental changes and grow quickly and densely. However, their fast growth rate depends on biotic factors, a differentiation in nutrients levels, global warming, and climate change [19,20].

Plant growth-promoting bacteria such as rhizospheric bacteria [21] and symbiotic rhizobia [22] are considered PHPB (plant health-promoting bacteria) agents. Additionally, in recent years, cyanobacteria have gained a great importance due to the fact that they have a significant role in regulating plant productivity [18]. In view of their benefits for soil fertility and crop production, this type of bacteria releases varied amounts of phytohormones (cytokinins, gibberellins, and auxins), amino acids, ammonia and polypeptides [23], siderophores [24], and polysaccharides [25] during active cell growth for plant growth and development [26]. These different types of substances represent important factors with stimulating effects on plant growth [27]. Notably, cyanobacterial biomass represents an effective bio-fertilizer source that plays an important role in improving soil physico-chemical characteristics such as the mineral nutrient status of the degraded lands and water-holding capacity [18]. Nowadays, cyanobacterial beneficial effects in the case of inoculation are reported in different crops such as cereals [28,29], pea plants [30], and romaine lettuce [31]. The application of cyanobacteria allows for the reduction of chemical fertilizer doses up to 50%, with a non-significant shortage in growth yield and plant biomass as a result for plant enrichment with auxin (IAA), cytokinins, gibberellic acids (Gibberellins), microelements (S, Zn, Fe, Mn, Cu, Mo, Co), amino acids, polyamines, macronutrients (N, P, K, Ca, Mg), and other secondary metabolites [32,33,34,35,36,37].

Traditionally, cyanobacterial species (i.e., *Nostoc* and *Anabaena*) are differentiated on the basis of morphological and life cycle characteristics. Even the 16S rRNA sequence for taxonomy is wide-spread in bacteria, and this technique represents a low variability in this region among species and strains [38]. *Nostoc* strains seem to form a monophyletic cluster that possibly contains more than one genus. The morphological features are stable and enough to separate different phylogenetic clusters (i.e., the length and width of akinetes are considered useful characteristics in classification of the *Anabaena*) [39].

In general, the application of cyanobacteria influences the spikelet opening angle, panicle exertion, and other floral traits, leading to a higher yield because of its ability to secrete Gibberellic acid (GA3). This can reflect on plant growth and thus on its productivity [35,40], growth, plant height, weight of 1000 grain, and grain yield of rice [41,42]. The present study aimed at: (1) isolating a new cyanobacterial isolate from the local area, (2) evaluating their production from IAA, and (3) exploring the influence of isolated cyanobacterial species such as *Anabaena* and *Nostoc* on enhancing floral traits and outcrossing rates in diverse cytoplasmic male sterile lines in rice and, subsequently, improving hybrid rice seed production.

## 2. Results

### 2.1. Cyanobacteria Isolation and Identification

**Figure 1:**
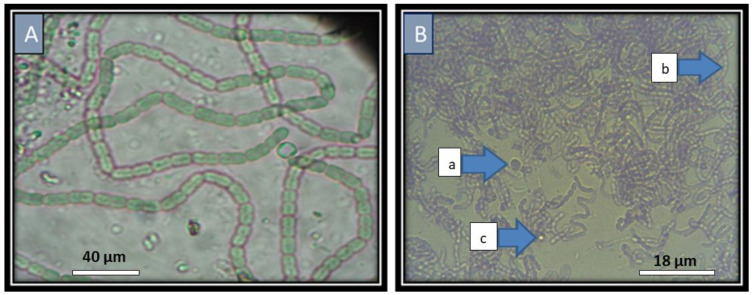
The two isolated cyanobacteria identified based on their morphological characteristics. (**A**) *Anabaena oryzae* with thallus green, which is gelatinous and memberanous. Trichomes are short and straight. Cells are barrel-shaped, the same length as it is broad, 4 μm broad, 5 μm long. Heterocysts are terminal and intercalary; intercalary heterocysts are subspherical, are 4–5 μm broad, and are 4–5 μm long; terminal heterocysts are conical and longer than they are broad, are 3–3.5 μm broad, and are 4–4.5 μm long. There are 3–6 akinetes in a series, and they are sub-spherical, are 5–6 μm broad, and are 6–7 μm long. (**B**) The *Nostoc muscorum* culture has dark green trichomes and no ramifications. It was uniseriate, single, aggregated, and showed neither polarity nor tapering. No sheath was formed. Trichomes are composed of three sizes and shapes of cells; (a) barrel cells (5–6 × 5.5–7 μm); (b) granular, ellipsoidal cells (5 × 5.5–7.5 μm); (c) yellowish-brown rounded cells of 8.9 μm in diameter. Few heterocysts were observed. They were of a single occurrence with two position, intercalary and terminal.

Cyanobacteria are dominant in soil. The collected samples from different sites in the Kafr El- Sheikh provenance were subjected to cyanobacteria isolation. Isolates were successfully obtained as free bacteria. The cyanobacteria were identified based on morphological characteristics as described in the method’s section. Two cyanobacteria were isolated and identified as *Anabaena oryzae* and *Nostoc muscorum* (Figure 1).

### 2.2. Production of Indole Acetic Acid (IAA)

The two selected cyanobacteria were subjected to IAA production measurement. Data in Table 1 indicated that IAA and its intermediates (i.e., indole-3-pyruvate (IPyA)) production increased gradually with cyanobacteria incubation. The continued inculcation with *Nostoc muscorum* gave higher values of IAA, which reached 4.4 times higher (37.01 µg/100 mL) after 21 days in comparison with the 7-day production (8.42 µg/mL), whereas lower values with *Anabaena oryzae* were recorded as 2.74, 11.15, and 11.74 μg/100 mL, respectively.

### 2.3. Total Cyanobacterial Count in Rice Field

Applying the inoculation of cyanobacteria (*N. muscorum* and *A. oryzae*) with CMS lines in soil was subjected to a total count in the rhizosphere. The changes in count during three periods (10, 35, and 70 days) after transplanting are presented in Table 2. The total number of inoculated treatments in the rhizosphere were higher than those in the un-inoculated (nil cyanobacterial) area. The counts gradually increased up to 850% and 650% in comparison with nil cyanobacterial treatment for *N. muscorum* and *A. oryzae,* respectively, 10 days after the inoculation. Notably, 260% and 180% increases in cell count were recorded with *N. muscorum* and *A. oryzae,* respectively, 35 days after treatment. After approximately 70 days, the count reflected noticeable changes, with 245% and 197% in *N. muscorum* and *A. oryzae* cells, respectively, when compared with untreated soil. In general, both cyanobacterial strains had a pronounced increase count in the three selected times.

### 2.4. Floral Traits

The assessed floral traits in CMS lines under cyanobacterial application and their interaction implied significant impacts of bacterial implementation on the measured floral traits. The application of T4 (mixture of the two cyanobacterial types), reflected the greatest enhancement and significant differences of all floral traits compared to T1 treatment (untreated control) (Table 3). L4 displayed the uppermost days to heading (DTH), the total stigma length (TSL), and the total stigma width (SW), while L5 possessed the highest spikelet opening angle (SOA) and duration of spikelet opening (DSO). Otherwise, the latest heading was presented by L3, while L4 recorded the lowest values of spikelet opening angle (SOA) and duration of spikelet opening (DSO). The lowest of TSL and SW were given by L5 and L1, respectively, during both seasons. The implementation of T4 enhanced SOA, DSO, TSL, and SW by 22.4%, 29.4%, 10.2%, and 18.4%, respectively, compared to the untreated control (T1) as an average of both seasons. Furthermore, the evaluated CMS lines recorded different responses to applied cyanobacteria (Table S2). The maximum days to heading, total stigma length, and total stigma width were assigned for L4 under T4 treatment while the highest spikelet opening angle and duration of spikelet opening were obtained by L5 under T4 treatment.

The influence of the interaction between cyanobacteria and CMS lines displayed highly significantly in days to heading (DTH), spikelet opening angle (SOA), duration of spikelet opening (DSO), total stigma length (TSL), and stigma width (SW) of CMS lines (Table S2). In general, the exogenous application of T4 (the combination between *A. Oryza* (T2) and *N. muscorum* (T3)) displayed the highest values of floral traits, followed by T3, T2, and then the untreated control, as an average of both years.

### 2.5. Growth-Related Traits

The evaluated CMS lines revealed that all growth-related traits were positively and significantly influenced by cyanobacteria treatment (Table 4). The CMS lines reflected different growth behavior; L4 exhibited the remarkable significant and higher values for the five growth traits on flag leaf area (FLA, 18.2%), plant height (PH, 9%), panicle length (PL, 7.8%), panicle exertion (PE, 10.7%), and leaf area index (LAI, 23%). On the other hand, L3 had the lowest values of growth traits (FLA, PH, PL, and LAI) compared to the other evaluated lines, whereas L2 displayed the lowest values of (PE) in both seasons. The impact of cyanobacteria, especially T4 treatment, recorded the maximum significant and positive values of the evaluated growth traits followed by T3 and T2 against untreated control. As an average of both years, T4 significantly increased flag leaf area (FLA, 23%), plant height (PH, 6.4%), panicle length (PL, 3,8%), panicle exertion (PE, 35.6%), and leaf area index (LAI, 8.8%), respectively, when compared to untreated lines (Table 4). Additionally, the interaction effect between cyanobacteria and CMS lines was highly significant on the flag leaf area, plant height, panicle length, panicle exertion, and leaf area index (Table S3). L4 under T4 applications exhibited the highest values for most growth traits, except the PE value, which was recorded in L1-T4. Otherwise, the lowest values for most traits were observed for the untreated plants of L3, except PE under L1-T1. The exogenous application of T4 displayed the highest and most significant values of all evaluated growth-related traits, except the leaf area index (cm), for which T4 exhibited the best values only for L3, L4, and L5.

### 2.6. Yield-Related Traits

As shown in Table 5, all yield-related traits were significantly influenced in CMS lines when the two cyanobacteria and their interaction were applied. The evaluated CMS lines reflected different growth behavior under the treatments, i.e., L4 exhibited remarkable higher positive and more significant values for the five measured traits (number of fertile panicles per hill (NFPH, 14%), panicle weight (PW, 18.2%), seed set (SS, 16.5%), grain yield (GY, 9.7%), and harvest index (HI, 11%)). The lowest NFPH was presented by L5, whereas L3 showed the latest values of PW, SS, GY, and HI in both seasons. The T4 treatment implied the uppermost values of NFPH, PW, SS, GY, and HI with percentages of 35.1, 90.3, 55.8, 77, and 22.4, respectively (Table 5). The influence of the interaction effect between cyanobacteria and CMS lines induced a highly significant effect on the number of fertile panicles per hill (NFPH), seed set (SS), and grain yield (GY) (Table S4). Eventually, T4 treatment displayed the highest values of NFPH, SS, and GY, and all tested CMS lines as well as L4–T4 recorded the best NFPH, SS, and GY values.

### 2.7. Relationships between CMS Lines, Bacterial Treatments, and Evaluated Traits

**Figure 2:**
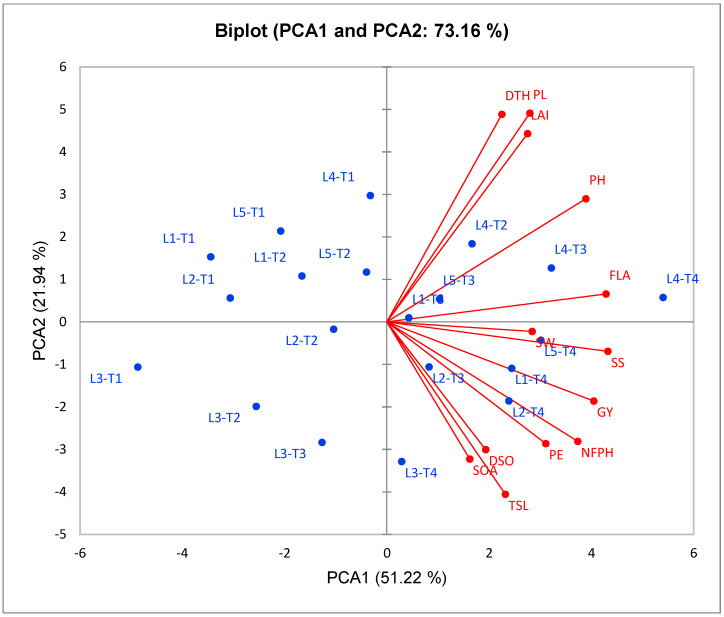
PC biplot for the applied cyanobacteria treatments and CMS lines over two growing seasons, depending on evaluated traits. DTH: days to heading, DSO: duration of spikelet opening, PE: panicle exertion, PW: panicle weight, SS: seed set, GY: grain yield, TSL: total stigma length, SOA: spikelet opening angle, SW: stigma width, LAI: leaf area index, NFPH: number of fertile panicles per hill, FLA: flag leaf angle, PH: plant height, PL: panicle length, and HI: harvest index.

The analyses of principal components was achieved to assess the relationship between CMS lines under cyanobacteria treatments. PCA1 and PCA2 revealed the most variability of the total variation with percentage 73.16% (Figure 2). Both PCA1 and PCA2 were used to build the PC-biplot. PCA2 exhibited a lower variation (21.94%) when compared to PCA1 (51.22%), which reflects a higher variation and seemed to correspond with CMS lines L3, L2, L1, L5, and L4 from top to bottom. In general, T1 and T2 were situated on the PCA1 negative side, T3 was located around the origin of the biplot, and T4 was presented on the PCA1 extremely positive side.

A robust positive relationship among the evaluated floral, growth, and yield traits and T4 were associated on the positive side of the PC1, in particular for L2, L1, and L5 lines, which indicates the high performance of these lines under T4 treatment in comparison with other treatments. On the contrary, the untreated CMS lines with cyanobacteria (control/T1 treatment), located on the opposite side, presented lower evaluated traits. Otherwise, both T2 and T3 existed between T1 and T4, presenting a slightly positive impact on the evaluated traits. The traits of floral, growth, and yield showed a highly significant positive inter-association by adjacent vectors, except for the following traits: flag leaf angle (FLA), plant height (PH), leaf area index (LAI), panicle length (PL), and days to heading (DTH). The traits SOA, DSO, and TSL were determined to be on the PCA2 positive side, while FLA and LAI existed on the negative side, which indicates a negative correlation between these traits.

The first three main PCAs gave the highest eigenvalues (6.658, 2.852, and 1.968, respectively), and they explained 88.29% (51.22%, 21.94%, and 15.14%, respectively) of the total variance of variables (Figure S2). Thus, PC1 and PC2 can be used as the basis for assessing the relationship between investigated traits under the main effect of the experimental factors. PC1 had a highly positive correlation with all studied traits. PC2 was strongly correlated with the spikelet opening angle, duration of spikelet opening, total stigma length, stigma width, panicle exertion, fertile panicles per hill, seed set, and grain yield (Table 6).

**Figure 3:**
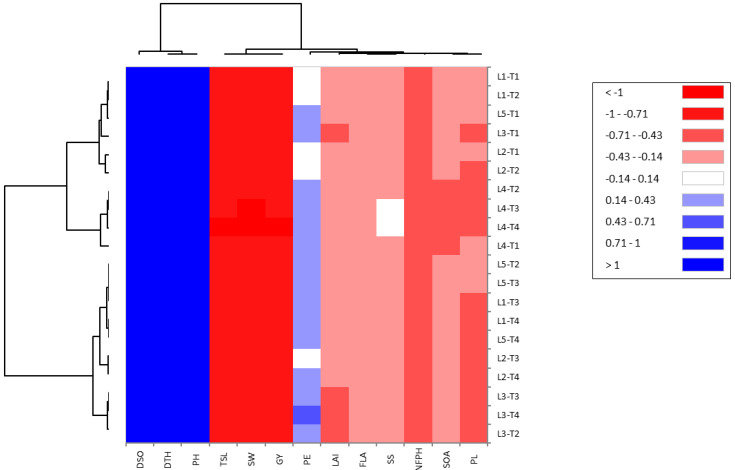
Correlation heatmap of the evaluated traits of CMS lines under cyanobacteria treatment conditions.

**Figure 4:**
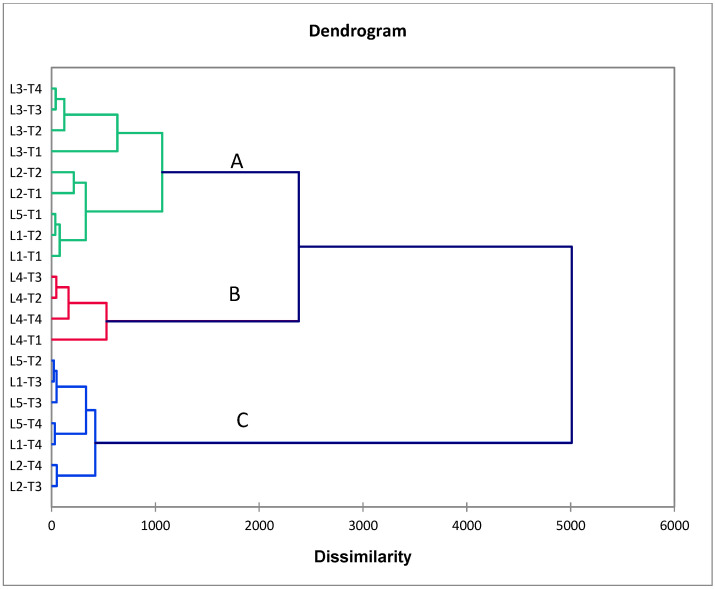
Agglomerative hierarchical clustering (AHC) based on the measured floral, growth, and yield traits divided the tested treatments into three main clusters.

Results depicted by the correlation heatmap divided the measured bacterial treatments and CMS lines into two main groups based on the evaluated traits, as shown in Figure 3. Treatments T3 and T4 were grouped together, whereas T1 and T2 were located in another cluster. A strong positive and significant association was detected among the duration of spikelet opening (DSO), days to heading (DTH), and plant height (PH). L3-T4 was considered the best value in panicle exertion (PE). Otherwise, a significant relationship was determined between total stigma length (TSL), stigma width (SW) and grain yield (GY) in the evaluated lines under bacterial treatments. On the other hand, the agglomerative hierarchical clustering (AHC) separated CMS lines with cyanobacteria treatments into three clusters (Figure 4). One cluster (cluster C) contained T3 and T4, while T1 and T2 located in cluster A.

## 3. Discussion

The CMS outcrossing rate was considered the main restricted reason in commercializing the hybrid rice. Therefore, enhancing the ability of outcrossing is necessary to increase hybrid seed production. Floral characters are very important and affect the efficacy of the outcrossing rate in CMS lines. Enhancing the CMS floral traits allows the opposition of foreign pollens, subsequently improving the process of cross-pollination and the production of the hybrid seed [43,44]. Cyanobacteria (blue-green algae) can fix atmospheric N in the wetland rice ecosystem and could significantly supply rice plants and soil with their need for nitrogen. The two genera *Nostoc* and *Anabaena* represent 80% of isolates in the rhizosphere. The two isolated cyanobacteria were identified based on their morphological characteristics. Makra et al [38] tried to determine the taxonomic relationships among *Anabaena* and *Nostoc* strains by 16S rRNA and *rbcLX* gene sequences. They found that some strains seemed to be identical when a comparison between them was performed by 16S rRNA or *rbcLX* sequence alignment.

The identified isolates are efficient in enhancing seed germination and growth in cereal crops such as rice and wheat via high protein accumulation in addition to IAA production [45,46,47]. The two isolated cyanobacterial strains produced up to 11.74 and 37.01 µg/100 mL of IAA and its intermediate compounds (i.e., indole-3-pyruvate (IPyA)) after 21 days of incubation. As reported, indole-3-pyruvate (IPyA) is the major intermediate and pathway for IAA biosynthesis in *Rhizobium tropici* CIAT 899 [48]. After adding tryptophan to the growth medium of *Azospirillum brasilense* Sp7, indole-3-pyruvic acid was detected in the broth, indicating the function of an aminotransferase pathway in IAA synthesis [49].

In the present study, the implementation of solely cyanobacteria (T4) remarkably improved all evaluated floral traits compared to the untreated control (T1). By applying T4 treatment (hybrid inoculum from *Anabaena Oryzae* and *Nostoc muscorum*), CMS lines had a longer duration of spikelet opening, a wider spikelet opening angle, a longer stigma, a wider stigma, and a higher stigma brush. Increasing the size of floret organs and the duration of the floret opening are favored features in out-crossing pollination. In this perspective, Zheng et al., Pathak et al., Majeed et al., and Kalavathi et al. [50,51,52,53] disclosed positive impacts of GA3, IAA, and NAA (Naphthalene acetic acid) on floral traits. Furthermore, the evaluated CMS lines exhibited highly significant variations in all evaluated floral traits. L4 and L1 displayed the uppermost evaluated floral traits, particularly under T4. Similar genotypic differences among CMS lines in floral traits were detected by El Sabagh et al. and Zheng et al. [8,43,54].

The enhancement of plant vegetative growth considerably reinforced hybrid seed production. The cyanobacterial mixture significantly enhanced flag leaf angle, plant height, panicle length, and panicle exertion of all evaluated CMS lines. The growth regulators produced by cyanobacteria enhance vegetative growth by boosting cell division, cell elongation, cell differentiation and enlargement, protein synthesis, content of chlorophyll, and efficiency of photosynthesis [55,56]. In Egypt, a study carried out by Salama [57] reported an increase in rice plant heights with values of 86.92 cm and 83.75 cm against the control (70.21 cm) after applying a treatment with *Nostoc* sp., *Anabaena* sp., and *Calothrix* sp. Additionally, findings indicated that individual or combinations of these cyanobacteria significantly increased the crop yield and 1000 grain weight of rice.

Poor panicle exertion of CMS lines considers a serious limitation in hybrid seed production. In this respect, the exogenous cyanobacterial mixture exhibited a significant increase in panicle length, panicle exertion, spikelet opening angle, duration of spikelet opening, total stigma length, stigma width, flag leaf angle, and leaf area index compared to untreated plants. Similar positive and significant impacts of growth regulators (cyanobacteria produce IAA) on panicle length, panicle exertion, and outcrossing rate were disclosed by Abo-Youssef et al., Hasan et al., and Pin et al. [58,59,60]. Furthermore, Gadallah [61]; Pal, et al. [62]; Hussain et al. (2021) [56] depicted that the application of growth regulators enhanced the plant height, panicle exertion, panicle length, and flag leaf angle of CMS lines. In the same manner, CMS lines exhibited highly significant differences in the genetic behavior for growth traits. L4 and L1 displayed the highest flag leaf angle, panicle length, plant height, and panicle exertion compared to the other evaluated CMS lines, particularly under the T4 treatment.

The total cyanobacterial count increased in all growth stages of nitrogen fertilization in rice plants [49]. The applied mixed cyanobacteria enhanced plant growth, which was reflected positively in seed yield and its contributing traits. This treatment reflected the highest improvement in panicle weight, grain yield, number of fertile panicles per hill, seed set, and harvest index. In light of that, Tiwari et al. [63]; Biswas et al. [64]; Pal et al. [62]; Hussain et al. [56] manifested that growth regulators boosted grain yield and its contributing traits in CMS lines.

Both cyanobacteria are plasticity and their ability to fix nitrogen allows the reduction of inorganic atmospheric nitrogen (N_2_) into organic nitrogen. The formed organic nitrogen is an ideal source of biofertilizer for plants. As a photosynthetic, cyanobacteria can also form symbiotic associations with other microorganisms and plants, subsequently using carbon dioxide (CO_2_) and water to generate monosaccharides and oxygen, which the plants can use [22]. Furthermore, cyanobacteria work well as PGPR (plant growth-promoting rhizobacteria) biofertilizers with PGP (plant growth promoting) traits, such as plant growth hormone production, nutrient solubilization, and siderophore production, which act as an antagonism to pathogenic fungi. Notably, they produce a source of bioactive molecules such as the diversity of secondary metabolites, vitamins, insecticides, immune suppressive activities, and herbicides [35].

To estimate the association between studied traits, the PCA biplot is a suitable method for this purpose. Our results from this approach reinforced the abovementioned results. Robust positive associations were identified among all evaluated floral and growth traits with yield traits in CMS lines under cyanobacteria treatment. Our findings confirmed that the application of T4 treatment with promising CMS lines, such as L4 and L1, enhances floral traits, plant growth, and plant productivity and hence increases the outcrossing rate and hybrid seed production in CMS lines.

## 4. Materials and Methods

### 4.1. Plant Materials and Treatments

Five CMS lines, three from the International Rice Research Institute (IRRI) as well as two from China, were selected for this study (the pedigree of utilized lines is presented in Table 7) based on their variable genetics. The following four cyanobacteria treatments were applied: (1) untreated control (T1), (2) *Anabaena Oryzae* (T2), (3) *Nostoc muscorum* (T3), and (4) a combination of *Anabaena Oryza* and *Nostoc muscorum* (T4).

A split-plot design with three replications was employed in the experiment. The CMS lines were located in the main plots, whereas cyanobacterial applications were laid out in sub-plots. Seedlings of CMS lines were hand transplanted with a row ratio of 8A:2B, 15 cm between rows of A lines, 20 cm between both B and A lines, 15 cm between hills (A and B lines), and 30 cm between rows of B lines, at the age of 30 days into hills with two seedlings per hill. Supplementary pollination was performed artificially by shaking the pollen parent’s canopy with a stick during flowering to spread pollen grains of B lines. The pollination process was carried out three times a day at 30-min intervals.

### 4.2. Experimental Site and Agronomic Treatments

The field experiment was performed during the summer of 2020 and 2021 at the experimental farm of Sakha Agricultural Research Station, Kafr El-Sheikh, Egypt (30°57′ N, 31°07′ E) to investigate the floral, growth, and yield traits of five diverse CMS lines. The temperature at the experimental site in the summer season ranged from 30 to 35 °C, with no precipitation events (representative of the Egypt summer season) during rice growing seasons. The soil of the experimental site with regard to physical and chemical properties and the meteorological is are presented in Table S1 and Figure S1. Phosphorus fertilizer at 36 kg was applied per hectare in superphosphate form (15.5% P_2_O_5_) as soil basal application during soil preparation. Nitrogen fertilizer at a rate of 124 kg ha^–1^ (75% from recommended doses) was added as urea (46% N). Two thirds of the N fertilizer were performed at the time of soil preparation for planting as a soil basal application, whereas the other one third was applied at panicle initiation. Moreover, zinc sulphate (22% ZnSO_4_) was implemented after paddling and before transplanting at the rate of 50 kg ha^−^^1^.

### 4.3. Cyanobacteria Isolation and Laboratory Studies

Soil samples were collected from the depth of 0–8 cm on the paddy field farm of Sakha Agricultural Research Station, Kafr El-Sheikh, Egypt. Cyanobacteria was isolated from dry soil samples as described by El-Ayouty and Ayyad [65] as follows: (1) Sterilized 0.7%, agarized, and modified Watanabe medium was poured into Petri dishes (10 cm in diameter). A few grams of soil samples were spread in the form of a strip (1 cm broad) in each Petri dish, then dishes were incubated at 30 °C under the continuous light of 120 cm-long white fluorescent lamps with a light intensity of 2500 Lux. (2) Soil samples were placed in 250 mL Erlenmeyer flasks containing 100 mL of sterilized liquid medium and left under the previously mentioned conditions of light and temperature. (3) The purification process of cyanobacteria was performed as follows: The unialgal cultures were purified as described by Pringsheim [66]. In all cases, any colored growth was picked up, sub cultured, and streaked several times in the new agarized medium BG11 plates. The previous procedure was repeated many times to obtain unialgal cultures. (4) Bacteria-free cultures from cyanobacteria: To obtain bacteria free cultures, serial attempts from the washing process with sterilized distilled water, as described by Hoshaw and Rosowski [67], were achieved as follows:

- Algal materials were placed in sterilized centrifuge tubes filled with sterilized distilled water.
- Algal materials were centrifuged at 3000 rpm for 10 min.
- The supernatant was decanted, and the washing process and centrifugation were repeated six times.
- The loosely packed algae were transferred after dilution into flasks containing fresh liquid BG11 medium and were incubated at 30 °C under the continuous light of 120 cm-long white fluorescent lamps with a light intensity that reached 2500 Lux for growth. Repeating the transfer process of algal units through distilled water or liquid medium followed by centrifugation may accomplish purification.

### 4.4. Identification of the Isolated Cyanobacteria:

Fourteen cyanobacteria isolates inoculated 500 mL Erlenmeyer flasks, each containing 250 mL of BG11 liquid medium and plates of BG11 solid medium with a loop full of 10-day-old culture. Inoculated flasks and plates were incubated at 28–30 °C under continuous illumination (2500 lux) for 10 days. Cyanobacteria identification was carried out using the following criteria: thallus morphology and dimension, thallus color, size of heterocyst, and vegetative and reproductive cells. In addition, heterocyst-forming cyanobacteria were also cultured in nitrogen-free Z medium [66,68]. Two cyanobacterial strains, *Anabaena Oryzae* and *Nostoc muscorum*, were identified and implemented in the current study.

### 4.5. Preparation of Standard Cyanobacterial Inoculum for Field Experiment

The inoculum of the identified cyanobacterial strains was prepared by culturing 500 mL Erlenmeyer flasks, each containing 250 mL of modified BG11 liquid medium, with a loopful of 21-day-old cultures of each strain. The inoculated flasks were incubated at 28–30 °C under continuous illumination (2500 lux) for 21 days. Furthermore, the growing inoculated flasks were incubated under artificial illumination (5000 lux) for one more month before field application. Inocula were prepared using a sieved clay soil before field application by mixing 100 mL of homogenous algal growth with an amount of 1 kg as a carrier. The mean number of algal cells in the inoculants was determined using the MPN method, as recommended by Cochran [69]. The cyanobacteria application was carried out 5 days after transplanting. All plots received the same agronomic treatments in terms of cultivation, seed rate, sowing methods, NPK and zinc fertilizers, weeds, and disease control. Nile tributary water was then introduced when height level reached five to ten cm, followed by further soil leveling under water [70].

### 4.6. Total Cyanobacterial Count

Soil samples were taken 10, 35, and 70 days after transplanting (DAT) to determine the total cyanobacterial count. *N. muscorum* and *A. oryzae* strains were counted on medium BG11 according to Ellora Malakar et al. [71]. Using the most probable number (MPN) technique, tubes were incubated at 30 °C under continuous light (white fluorescent lamps, 120 cm long, and 2500 lux intensity).

### 4.7. Production of IAA

According to Ahmad et al. [72], each bacterial strain was grown in its specific medium supplemented with 0.1% tryptophane. IAA production in the supernatant was measured via the PC method, according to the Salkowski colorimetric technique as described by Pilet and Chollet [73,74]. Briefly, the reaction contained 1 mL of reagent (FeCl_3_, 12 g/L dissolved in 7.9 M H_2_SO_4_) mixed with 1 mL of the sample supernatant and left in the dark at room temperature for 30 min. Absorbance at 530 nm was measured, and IAA concentrations were calculated from the generated IAA standard curve.

### 4.8. Measured Traits

For the studied characters, data were recorded from five randomly selected hills, excepting border rows per sub-plot. The floral traits were measured using a micrometer under a stereomicroscope according to Singh and Haque [75]. The floral traits under study involved total stigma length (mm), stigma width (mm), duration of spikelet opening (min), spikelet opening angle (°), and days to heading (50%). Furthermore, ten randomly selected plants from each plot were subjected to growth and yield trait measurement, including panicle length (cm), panicle exertion (%), plant height (cm), flag leaf angle (°), leaf area index (cm), number of fertile tillers per hill (n), seed set (%), and seed yield (ton ha^−1^). When 85% of the grains became golden yellow, the crop was harvested, and to estimate grain yield, grains were sun-dried and moisture content was adjusted to 14%.

$$\mathrm{Panicle}\;\mathrm{exertion}\%=\frac{\mathrm{Length}\;\mathrm{of}\;\mathrm{exserted}\;\mathrm{part}\;\mathrm{of}\;\mathrm{panicle}\;\left(\mathrm{cm}\right)\;}{\mathrm{whole}\;\mathrm{Panicle}\;\mathrm{length}\;\left(\mathrm{cm}\right)}\times 100$$

$$\mathrm{Seed}\;\mathrm{set}\%=\frac{\mathrm{Number}\;\mathrm{of}\;\mathrm{filled}\;\mathrm{grains}/\mathrm{panicle}}{\mathrm{Total}\;\mathrm{number}\;\mathrm{of}\;\mathrm{spikelets}/\mathrm{panicle}}\times 100$$

### 4.9. Statistical Analysis

The data obtained were analyzed using analysis of variance (ANOVA) following the standard procedure given by Gomez and Gomez [76], using DSTAAT computer software. The mean differences between treatments were measured by Duncan’s Multiple Range Test (DMRT) at the level of *p* = 0.05. XLSTAT software version 2019 was used to carry out the PCA, biplot, and heatmap [77] to explore the relationship among the evaluated traits. PCs with eigenvalues greater than one were kept, but PCs with eigenvalues less than one were eliminated [78]. Plant variable groups (dendrogram) were established using a technique called agglomerative hierarchical clustering (AHC). The data were divided into many clusters with a common trait using the XLSTAT software version 2019.

## 5. Conclusions

Cyanobacteria application, in combination with CMS lines in rice, remarkably ameliorated all evaluated floral, growth, and yield characteristics. Additionally, the evaluated CMS lines exhibited different genetic behaviors for floral traits, plant growth, and plant productivity. Different plant traits, comprising floral, plant growth, and grain yield traits, in L4 and L5 exhibited the uppermost values, particularly under T4 treatment. Consequently, it seems interesting to exploit this mixing of cyanobacteria with the promising CMS lines L4, L1, and L5 as a useful tool in enhancing the outcrossing rates and improving the seed production of hybrid rice.

## Figures and Tables

**Table 1:** The mean production of IAA from the tested cyanobacterial strains (μg/100 mL) ± standard deviation.

Cyanobacteria Strains	Culture Age Days		
	7	14	21
*Anabaena oryzae*	2.74 ± 0.09	11.15 ± 1.04	11.74 ± 0.06
*Nostoc muscorum*	8.42 ± 1.12	21.96 ± 1.28	37.01 ± 1.32
LSD 0.05	1.6	1.64	1.79

LSD = least significant difference.

**Table 2:** Average total cyanobacterial count (10^4^ cfu g^−1^ dry soil) in the rhizosphere of rice plant at three periods after transplanting ± standard deviation.

Treatment	Days after Transplanting		
	10	35	70
*Nil cyanobacterial strains*	0.010 + 0.004	0.074 + 0.010	0.095 + 0.016
*Nostoc muscorum*	0.085 + 0.006	0.192 + 0.007	0.232 + 0.018
*Anabaena oryzae*	0.065 + 0.009	0.133 + 0.007	0.187 + 0.011

**Table 3:** The impact of two isolated cyanobacteria and their mixture on days to heading (DTH), spikelet opening angle (SOA), duration of spikelet opening (DSO), total stigma length (TSL), and stigma width (SW) of CMS lines. The table represents the mean performance of CMS lines under all treatments (upper part) and the mean performance of treatments in all CMS lines (down part) with regard to measured floral traits.

Studied Factor	DTH (50%)		SOA (°)		DSO (min)		TSL (mm)		SW (mm)	
	2020	2021	2020	2021	2020	2021	2020	2021	2020	2021
**CMS line (L)**										
L1 (A1 × B1)	106.84 _b_	108.13 _b_	25.62 _a_	27.49 _a_	140.23 _b_	146.48 _b_	1.47 _c_	1.52 _c_	0.31 _e_	0.33 _e_
L2 (A2 × B2)	90.71 _c_	92.00 _c_	24.97 _b_	26.84 _b_	138.71 _c_	144.96 _c_	1.57 _b_	1.61 _b_	0.40 _d_	0.42 _d_
L3 (A3 × B3)	89.06 _c_	90.35 _c_	22.95 _c_	24.82 _c_	127.29 _d_	133.54 _d_	1.58 _a_	1.63 _a_	0.42 _c_	0.44 _c_
L4 (A4 × B4)	110.19 _a_	111.48 _a_	20.63 _d_	22.50 _d_	114.71 _e_	120.96 _e_	1.59 _a_	1.64 _a_	0.49 _a_	0.51 _a_
L5 (A5 × B5)	105.16 _b_	106.45 _b_	25.46 _a_	27.33 _a_	148.23 _a_	154.48 _a_	1.44 _d_	1.48 _d_	0.43 _b_	0.45 _b_
**Treatment (T)**										
T1	99.48 _d_	100.77 _d_	21.53 _d_	23.40 _d_	114.46 _d_	120.71 _d_	1.45 _d_	1.49 _d_	0.37 _d_	0.39 _d_
T2	100.10 _c_	101.39 _c_	22.76 _c_	24.63 _c_	130.93 _c_	137.18 _c_	1.51 _c_	1.55 _c_	0.41 _c_	0.43 _c_
T3	100.72 _b_	102.01 _b_	24.85 _b_	26.72 _b_	140.95 _b_	147.20 _b_	1.55 _b_	1.59 _b_	0.42 _b_	0.44 _b_
T4	101.26 _a_	102.55 _a_	26.56 _a_	28.43 _a_	149.00 _a_	155.25 _a_	1.60 _a_	1.64 _a_	0.44 _a_	0.46 _a_
T × L	**	**	**	**	**	**	**	**	**	**

T1: untreated control, T2: *A. Oryzae,* T3: *N. muscorum*, T4: the combination between T2 and T3. Different lowercase letters within a column indicate a significant difference among the CMS lines under each treatment at *p* < 0.05. ** = highly significant. T × L = interaction between treatments and CMS lines.

**Table 4:** The impact of cyanobacteria application on the flag leaf angle (FLA), plant height (PH), panicle length (PL), panicle exertion (PE), and leaf area index (LAI) of CMS lines. The table represents the mean performance of CMS lines under all treatments (upper part) and the mean performance of treatments in all CMS lines (lower part) regarding growth-related traits.

Studied Factor	FLA (°)		PH (cm)		PL (cm)		PE (%)		LAI (cm)	
	2020	2021	2020	2021	2020	2021	2020	2021	2020	2021
**CMS lines (L)**										
L1 (A1 × B1)	27.75 _c_	26.94 _c_	98.52 _c_	100.57 _c_	22.03 _b_	23.32 _b_	50.30 _b_	48.45 _b_	30.36 _c_	27.86 _c_
L2 (A2 × B2)	28.61 _b c_	27.79 _b c_	100.97 _b_	103.02 _b_	20.72 _b_	22.01 _b_	46.53 _c_	44.67 _c_	33.22 _b_	30.73 _b_
L3 (A3 × B3)	26.44 _d_	25.63 _d_	92.76 _d_	94.81 _d_	18.36 _c_	19.65 _c_	55.65 _a_	53.71 _a_	17.49 _d_	14.99 _d_
L4 (A4 × B4)	32.72 _a_	31.90 _a_	107.57 _a_	109.62 _a_	23.84 _a_	25.05 _a_	54.71 _a_	52.65 _a_	37.06 _a_	34.56 _a_
L5 (A5 × B5)	29.54 _b_	28.73 _b_	101.46 _b_	103.51 _b_	23.76 _a_	25.13 _a_	55.60 _a_	53.75 _a_	30.94 _c_	28.44 _c_
**Treatment (T)**										
T1	26.15 _d_	25.32 _d_	97.23 _d_	99.28 _d_	21.29 _d_	22.58 _d_	45.04 _d_	43.18 _d_	28.40 _d_	25.90 _d_
T2	28.27 _c_	27.46 _c_	99.07 _c_	101.12 _c_	21.62 _c_	22.91 _c_	49.91 _c_	48.05 _c_	29.96 _c_	27.36 _c_
T3	29.55 _b_	28.74 _b_	101.21 _b_	103.26 _b_	21.93 _b_	23.22 _b_	54.31 _b_	52.45 _b_	30.22 _b_	27.72 _b_
T4	32.07 _a_	31.26 _a_	103.52 _a_	105.57 _a_	22.13 _a_	23.41 _a_	60.74 _a_	58.89 _a_	30.78 _a_	28.28 _a_
T × L	**	**	**	**	**	**	**	**	**	**

Untreated control (T1), *A. Oryzae* (T2), *N. muscorum* (T3), and their combination (T4). Different lowercase letter within a column indicates significant difference among the CMS lines under each treatment at *p* < 0.05, ** = high significant. T × L = Interaction between treatments and CMS Lines.

**Table 5:** The impact of cyanobacteria on the number of fertile panicles per hill (NFPH), panicle weight (PW), seed set (SS), and grain yield (GY) of CMS lines. The table represents the mean performance of CMS line yield-related traits under all treatments (upper part) and the mean performance of treatments in all CMS lines (down part) regarding yield-related traits. T1: untreated control, T2: *A. Oryzae*, T3: *N. muscorum*, T4: the combination between T2 and T3. Different lowercase letters within a column indicate a significant difference among the CMS lines under each treatment at *p* < 0.05. ** = highly significant, NS = not significant. T × L = interaction between treatments and CMS lines.

Studied Factor	NFPH		PW (g)		SS (%)		GY (ton/ha)		HI (%)	
	2020	2021	2020	2021	2020	2021	2020	2021	2020	2021
**CMS lines (L)**										
L1 (A1 × B1)	15.54 _c_	17.52 _c_	1.98 _b_	2.24 _b_	32.05 _c_	30.76 _c_	0.868 _b_	1.020 _b_	16.05 _b_	17.34 _b_
L2 (A2 × B2)	16.84 _b_	18.82 _b_	1.73 _d_	1.98 _d_	30.05 _d_	28.75 _d_	0.901 _b_	1.054 _b_	14.60 _d_	15.89 _d_
L3 (A3 × B3)	16.05 _c_	18.03 _c_	1.63 _e_	1.88 _e_	28.72 _e_	27.42 _e_	0.715 _c_	0.867 _c_	13.70 _e_	14.99 _e_
L4 (A4 × B4)	17.86 _a_	19.84 _a_	2.37 _a_	2.62 _a_	37.25 _a_	35.95 _a_	0.959 _a_	1.112 _a_	17.90 _a_	19.19 _a_
L5 (A5 × B5))	14.74 _d_	16.72 _d_	1.89 _c_	2.14 _c_	32.62 _b_	31.33 _b_	0.758 _c_	0.911 _c_	15.94 _c_	17.23 _c_
**Treatment (T)**										
T1	13.74 _d_	15.72 _d_	1.26 _d_	1.51 _d_	25.05 _d_	23.76 _d_	0.581 _d_	0.733 _d_	14.08 _d_	15.37 _d_
T2	15.32 _c_	17.30 _c_	1.79 _c_	2.04 _c_	30.17 _c_	28.88 _c_	0.753 _c_	0.905 _c_	14.97 _c_	16.26 _c_
T3	16.84 _b_	18.82 _b_	2.14 _b_	2.39 _b_	34.65 _b_	33.36 _b_	0.940 _b_	1.093 _b_	16.12 _b_	17.41 _b_
T4	18.91 _a_	20.89 _a_	2.51 _a_	2.76 _a_	38.67 _a_	37.38 _a_	1.087 _a_	1.239 _a_	17.38 _a_	18.67 _a_
T × L	**	**	NS	NS	**	**	**	**	NS	NS

**Table 6:** Results of principal component analysis (PCs) in the first five PCs for the studied traits during the main effects of experimental factors.

Variables	PC1	PC2	PC3	PC4	PC5
Days to heading	0.196	−0.425	0.148	0.296	−0.494
Spikelet opening angle (°)	0.141	0.280	0.557	−0.105	0.134
Duration of spikelet opening (min)	0.168	0.261	0.527	0.011	0.333
Total stigma length (mm)	0.201	0.352	−0.365	−0.252	−0.168
Stigma width (mm)	0.247	0.020	−0.432	0.276	0.603
Flag leaf angle (%)	0.373	−0.057	−0.081	0.020	0.108
Plant height (cm)	0.338	−0.252	−0.069	−0.184	0.151
Panicle length (cm)	0.243	−0.427	0.169	0.147	0.145
Panicle exertion (PE) (%)	0.270	0.249	0.013	0.623	−0.079
Leaf area index (cm)	0.239	−0.385	0.056	−0.458	0.153
Fertile panicles per hill	0.325	0.244	−0.150	−0.192	−0.195
Seed set (%)	0.376	0.060	0.023	0.117	−0.221
Grain yield (tons/ha)	0.352	0.162	0.058	−0.239	−0.254
Eigenvalue	6.658	2.852	1.968	0.835	0.438
% variance	51.218	21.935	15.14	6.421	3.371
Cumulative% variance	51.218	73.153	88.293	94.714	98.085

**Table 7:** Cytoplasmic male sterile (CMS) lines used in the current study.

Code	CMS Line	CMS Line Code	Maintainer	Maintainer Code	Cytoplasmic Source	Origin
L1	IR69625A	A1	IR69625B	B1	Wild abortive (WA) CMS line	IRRI
L2	IR58025A	A2	IR58025B	B2	Wild abortive (WA) CMS line	IRRI
L3	IR70368A	A3	IR70368B	B3	Wild abortive (WA) CMS line	IRRI
L4	G46A	A4	G46B	B4	Gambiaca CMS line	China (Hunan Academy of Agricultural Sciences)
L5	K17A	A5	K17B	B5	Kalinga type	China (Hunan Academy of Agricultural Sciences)

## Data Availability

The data presented in this study are available upon request from the corresponding author.

## Supplementary Materials

The following supporting information can be downloaded at: https://www.mdpi.com/article/10.3390/plants11243411/s1, Figure S1: Certain meteorological data at the experimental site in the two summer seasons of 2020 and 2021; Figure S2: Scree plot of PCA between respective eigenvalues %, components number and cumulative variability (%); Table S1: Physical and chemical soil characteristics at the experimental site, during 2020 and 2021 growing seasons; Table S2: Influence of interaction between cyanobacteria and CMS lines on days to heading (DTH), spikelet opening angle (SOA), duration of spikelet opening (DSO), total stigma length (TSL) and stigma width (SW) of CMS lines; Table S3: Influence of interaction between application of cyanobacteria and CMS lines on flag leaf angle (FLA), plant height (PH), panicle length (PL), panicle exertion (PE) and leaf area index (LAI) of CMS lines; Table S4: Influence of interaction between the application of cyanobacteria and CMS lines on number of fertile panicles per hill (NFPH), seed set (SS) and grain yield (GY) of CMS lines
