# Supplementary material for: Cyanobacteria Application Ameliorates Floral Traits and Outcrossing Rate in Diverse Rice Cytoplasmic Male Sterile Lines
Source: Plants (Basel). 2022 Dec 7;11(24):3411. doi: 10.3390/plants11243411 (PMC9781212; doi:10.3390/plants11243411)
Supplement: Supplementary file 1 [file plants-11-03411-s001.zip › plants-1938387-supplementary.pdf]

Table S1. Physical and chemical soil characteristics at the experimental site, during 2020 and 2021 growing seasons

**Characteristics**	**2020**	**2021**
Soil particles distribution		
Sand (%)	15.35	14.78
Silt (%)	33.18	32.47
Clay (%)	51.48	52.71
Soil texture	Clayey	Clayey
pH (1: 2.5 water suspension)	8.04	8.16
EC (dS m^{-1})	2.29	2.55
Organic matter	1.64	1.57
Soluble cations and anions (mmolc L^{-1})		
Ca^{2+}	10.69	10.1
Mg^{2+}	4.22	3.95
K^{+}	1.93	1.80
Na^{+}	14.00	13.10
HCO_{3}^{-}	13.60	15.50
Cl^{-}	8.50	7.40
SO_4^{2-}	8.60	7.60
CO_3^{2-}	0.0	0.0

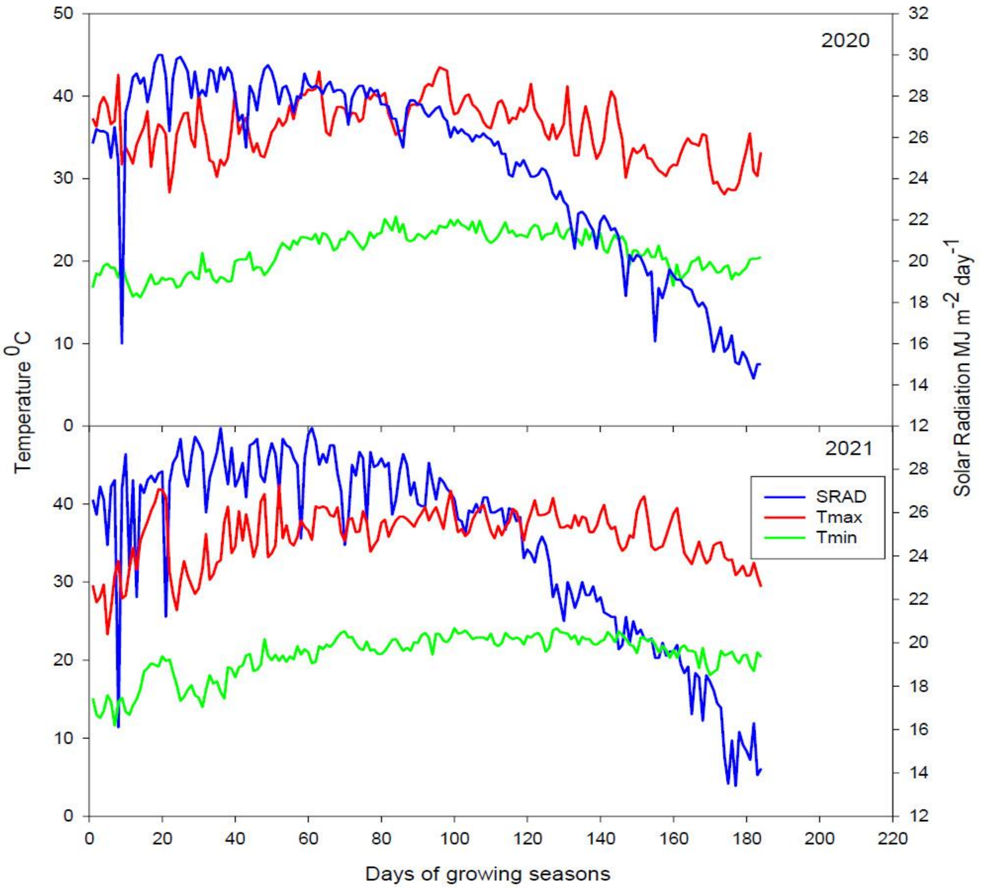

Figure S1. Certain meteorological data at the experimental site in the two summer seasons of 2020 and 2021.

Table S2. Influence of interaction between cyanobacteria and CMS lines on days to heading (DTH), spikelet opening angle (SOA), duration of spikelet opening (DSO), total stigma length (TSL) and stigma width (SW) of CMS lines

CMS line	Treatment	DTH (50%)		SOA (^{0})		DSO (min)		TSL (mm)		SW (mm)	
		2020	2021	2020	2021	2020	2021	2020	2021	2020	2021
L1 (A1×B1	T1	105.46k	106.75j	23.07h	24.94g	125.45m	131.70m	1.44n	1.48n	0.26o	0.28o
	T2	106.40g	107.75g	24.24f	26.11f	129.63j	135.88j	1.45m	1.49m	0.30n	0.32n
	T3	107.36f	108.65f	26.57d	28.44d	145.36g	151.61g	1.48l	1.52l	0.32m	0.34m
	T4	108.06e	110.48d	28.59a	30.46a	160.49b	166.74b	1.54i	1.57i	0.35l	0.37l
L2 (A2×B2)	T1	89.89p	91.18p	22.63hi	24.52hi	114.41o	120.66o	1.52j	1.55j	0.37j	0.39j
	T2	90.39o	91.68o	23.87fg	25.74fg	133.62i	139.86i	1.56g	1.60g	0.39i	0.42i
	T3	90.99n	92.28n	25.77e	27.64e	151.62e	157.86e	1.59e	1.63e	0.41h	0.43h
	T4	91.59m	92.88m	27.58b	29.45b	155.22c	161.47c	1.60e	1.64e	0.43e	0.45e
L3 (A3×B3)	T1	88.49t	89.78t	20.75m	22.62m	108.76p	115.01p	1.50k	1.54k	0.36k	0.38k
	T2	88.79s	90.08s	22.02k	23.89k	128.60k	134.85j	1.58f	1.62f	0.42f	0.44f
	T3	89.29r	90.58r	23.58g	25.45g	133.25i	139.50i	1.62d	1.66d	0.43e	0.45e
	T4	89.69q	90.98p	25.44e	27.31e	138.55h	144.80h	1.65b	1.69b	0.45d	0.47d
L4 (A4×B4)	T1	109.19d	110.48d	19.00o	20.87o	98.75q	105.00q	1.43o	1.47o	0.45d	0.47d
	T2	109.99c	111.28c	19.66n	21.53n	113.75o	120.00o	1.58f	1.62f	0.48c	0.50c
	T3	110.49b	111.78b	21.32l	23.19l	120.16n	126.41o	1.64c	1.68c	0.50b	0.52b
	T4	111.09a	112.38a	22.56ij	24.43ij	126.18l	132.43l	1.68a	1.72a	0.53a	0.55a
L5 (A5×B5)	T1	104.38l	105.67l	22.18jk	24.05jk	124.91m	131.16m	1.36q	1.40q	0.41g	0.43g
	T2	104.88k	106.17k	24.02fg	25.89fg	149.06f	155.31f	1.41p	1.45p	0.42f	0.44f
	T3	105.48i	106.77i	27.03c	28.90c	154.36d	160.61d	1.45m	1.49m	0.43e	0.44f
	T4	105.88h	107.17h	28.63a	30.50a	164.58a	170.83a	1.55h	1.59h	0.45d	0.47d

T1: untreated control, T2: Anabaena Oryzae, T3: Nostoc muscorum, T4: the combination between T2 and T3. Different lowercase letter within a column indicates significant difference among the CMS lines within each growth regulator at $p < 0.05$.

Table S3. Influence of interaction between application of cyanobacteria and CMS lines on flag leaf angle (FLA), plant height (PH), panicle length (PL), panicle exertion (PE) and leaf area index (LAI) of CMS lines

CMS line	Treatment	FLA (^{o})		PH (cm)		PL (cm)		PE (%)		LAI (cm)	
		2020	2021	2020	2021	2020	2021	2020	2021	2020	2021
L1 (A1×B1)	T1	25.33h	24.52h	95.63q	97.68q	21.33l	22.62l	38.80t	36.95t	29.08f	26.58f
	T2	26.12g	25.31g	97.63o	99.68o	21.93k	23.22k	45.80q	43.95q	30.98def	28.48def
	T3	28.03e	27.52e	99.93l	101.98l	22.43j	23.72j	53.80j	51.95j	31.09def	28.59def
	T4	31.23c	30.42c	100.89h	102.94i	22.44i	23.73i	62.80a	60.95a	30.29ef	27.79ef
L2 (A2×B2)	T1	24.77h	23.90h	98.43m	100.48m	20.17p	21.46p	40.13s	38.27s	32.32c-f	29.82c-f
	T2	28.03e	27.22e	100.03k	102.08k	20.57o	22.22n	43.13r	41.27r	33.22b-f	30.72b-f
	T3	29.93d	29.12d	101.83h	103.88h	20.93n	22.22n	47.63o	45.77o	34.62a-e	32.12a-e
	T4	31.73c	30.92c	103.61f	105.66f	21.22m	22.51m	54.27i	53.37h	32.74b-f	30.24b-f
L3 (A3×B3)	T1	23.27i	22.46i	89.28t	91.34t	18.06t	19.35t	48.42n	46.57n	16.15g	13.64g
	T2	27.88e	27.07e	91.18s	93.23s	18.26s	19.55s	52.78l	50.93l	17.64g	15.15g
	T3	26.17g	25.36g	93.78r	95.84r	18.46r	19.75r	58.38e	56.53e	17.1g	14.65g
	T4	28.33e	27.66e	96.78p	98.84q	18.66q	19.95q	62.68b	60.83b	19.05g	16.54g
L4 (A4×B4)	T1	30.41d	29.61d	104.45e	106.50e	23.31h	24.60h	47.56p	45.70p	35.24a-d	32.74a-d
	T2	31.42c	30.62c	106.35c	108.40c	23.66f	24.95f	53.56k	51.71k	36.74abc	34.24abc
	T3	33.31b	32.51b	108.35b	110.40b	24.01b	25.30b	56.27f	54.42f	37.24ab	34.74ab
	T4	35.71a	34.91a	111.15a	113.20a	24.36a	25.65a	61.45d	59.61d	39.04a	36.54a
L5 (A5×B5)	T1	26.94f	26.13f	98.37n	100.42n	23.58g	24.87g	50.27m	48.43m	29.23f	26.73f
	T2	27.94f	27.13e	100.16j	102.21j	23.70e	24.99e	54.27i	52.43i	30.73def	28.23def
	T3	30.04d	29.23d	102.16g	104.21j	23.82d	25.30b	56.28f	54.53f	32.0def	28.50def
	T4	33.24b	32.43b	105.16d	107.20d	23.94c	25.23c	61.57c	59.73c	32.80b-f	30.30b-f

Table S4. Influence of interaction between the application of cyanobacteria and CMS lines on number of fertile panicles per hill (NFPH), seed set (SS) and grain yield (GY) of CMS lines

CMS line	Treatment	NFPH (n)		SS (%)		GY (ton/ha)	
		2020	2021	2020	2021	2020	2021
L1 (A1×B1)	T1	13.14m	15.12m	24.40m	23.11m	0.554s	0.706q
	T2	14.58k	16.56k	30.20h	28.91h	0.752l	0.904l
	T3	16.35g	18.33g	34.50e	33.21e	1.005e	1.157e
	T4	18.08c	20.06c	39.10c	37.81c	1.161b	1.313be
L2 (A2×B2)	T1	13.93l	15.92l	22.43n	21.14n	0.639o	0.791o
	T2	15.52i	17.49i	28.03j	26.74j	0.835j	0.987j
	T3	17.84cd	19.82cd	32.76f	31.47f	1.005e	1.157e
	T4	20.06a	22.04a	36.97d	35.68d	1.128c	1.280c
L3 (A3×B3)	T1	13.34m	15.32m	22.04n	20.75n	0.502s	0.654s
	T2	15.12j	17.10j	26.74l	25.45l	0.638p	0.790p
	T3	17.22f	19.20f	31.56g	30.27g	0.797k	0.949k
	T4	18.52b	20.50b	34.52e	33.23e	0.922g	1.074g
L4 (A4×B4)	T1	15.86h	17.84h	29.22i	27.92i	0.689n	0.841n
	T2	17.46ef	19.44ef	34.23e	32.93e	0.849i	1.001i
	T3	17.76cde	19.73cde	39.82b	38.53b	1.034d	1.186d
	T4	20.36a	22.34a	45.72a	44.43a	1.264a	1.416a
L5 (A5×B5)	T1	12.44n	14.42n	27.16k	25.87k	0.521r	0.673r
	T2	13.94l	15.92l	31.56g	30.27g	0.690m	0.843m
	T3	15.04j	17.02j	34.61e	33.32e	0.849h	1.013h
	T4	17.54def	19.52def	37.05d	35.68d	0.963f	1.114f

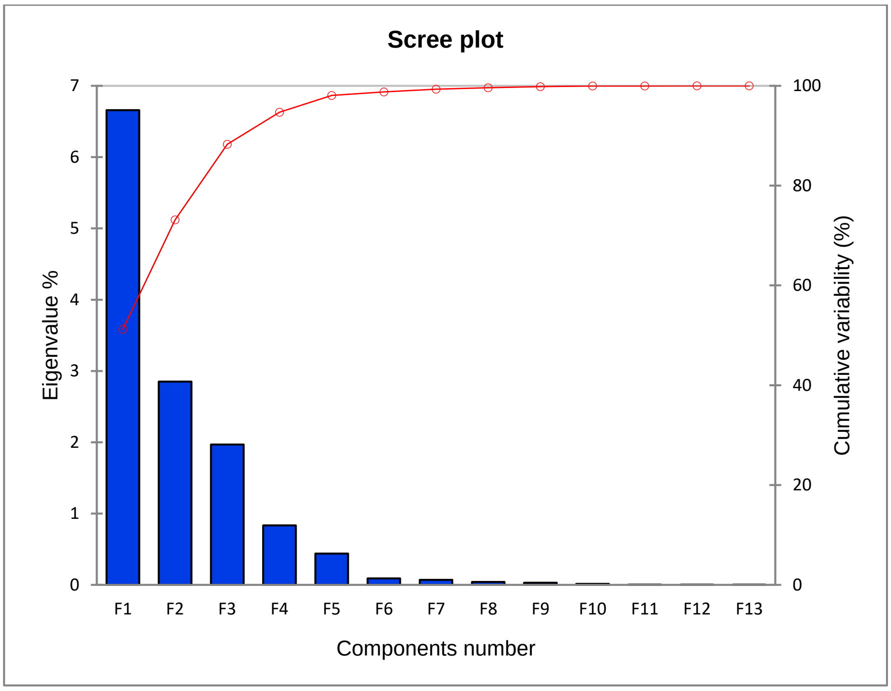

Figure S2. Scree plot of PCA between respective eigenvalues %, components number and cumulative variability (%).
